# Monitoring and Evaluating Progress towards Universal Health Coverage in Thailand

**DOI:** 10.1371/journal.pmed.1001726

**Published:** 2014-09-22

**Authors:** Viroj Tangcharoensathien, Supon Limwattananon, Walaiporn Patcharanarumol, Jadej Thammatacharee

**Affiliations:** 1 International Health Policy Program (IHPP), Ministry of Public Health, Nonthaburi Province, Thailand; 2 Khon Kaen University, Khon Kaen Province, Thailand; 3 National Health Security Office, Nonthaburi Province, Thailand

## Abstract

This paper is a country case study for the Universal Health Coverage Collection, organized by WHO. Walaiporn Patcharanarumol and colleagues illustrate progress towards UHC and its monitoring and evaluation in Thailand.

*Please see later in the article for the Editors' Summary*

This paper is part of the PLOS Universal Health Coverage Collection. This is the summary of the Thailand country case study. The full paper is available as Supporting Information file Text S1.

## Background

Before 2002, 30% of the entire Thai population of 63 million people remained uninsured. With the advent of the Universal Coverage Scheme (UCS) that combined a medical welfare low-income card scheme and a government-subsidized voluntary health card scheme with a coverage extension to the remaining uninsured, Thailand achieved the status of universal health coverage (UHC) in 2002 in terms of insurance entitlement, when the gross national income per capita was US$ 1,900 [1].

## Universal Health Coverage: The Policy Context

Two broad strands of health systems development were applied well before the UCS: (1) expansion of infrastructure and human resources to district health systems during the 1970s and 1980s and (2) extension of financial risk protection from 1975 onwards, targeting different groups of the population. This development resulted in high levels of health service utilization with narrow socio-economic gaps [2].

The tax-financed UCS is progressive. A close-end provider payment contributes to cost containment and systems efficiency. While it was financially feasible to offer a comprehensive benefit package at the launch of UCS in 2002, a few high-cost services–in particular anti-retroviral treatment and renal replacement therapy–were introduced later in 2003 and 2006 respectively [3].

## Monitoring and Evaluation of UHC

At the inception of the UCS, there were no explicit monitoring and evaluation (M&E) blueprints. Development of information systems and the M&E indicators have been incrementally built on existing platforms. A high coverage of civil registration–96.7% of births and 95.2% of deaths–facilitates UHC monitoring (Text S1). The sharing and interoperability of health insurance member databases across three schemes not only contributed to accurate headcounts but ensured entitlement to the covered health services.

Existing nationally representative household surveys, namely (1) the Health and Welfare Survey (HWS) and (2) the Socio-economic Survey (SES) and a long series of the National Health Account, are the main components of the assessment of the overall level and distribution of health utilization, benefit incidence, and financial risk protection. The National Health Examination Survey (NHES), locally initiated and financed, contributes to the measurement of effective coverage of key chronic non-communicable diseases (NCDs). Routine administrative data and various disease registries were established in response to program management requirements.

## Progress towards UHC in Thailand

Existing evidence demonstrates favourable UHC outcomes: equitable access to health services, a low level of unmet health needs, and a high level of financial risk protection. Since the early 2000s, all health Millennium Development Goals have been achieved reflecting the health systems' resilience and capacities. Thailand ranked first among 80 countries with the highest average annual reduction (8.5%) in child mortality between 1990 and 2006 [4].

**Figure 1:**
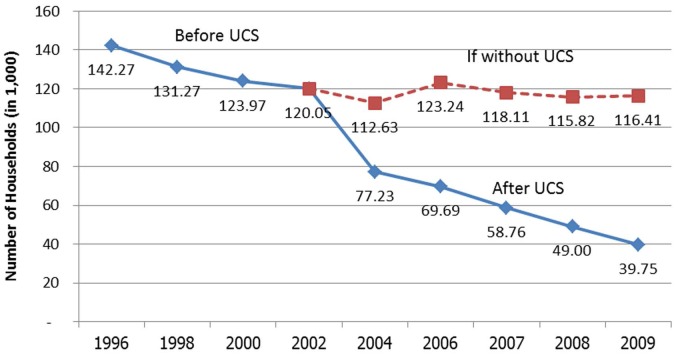
Number of households prevented from medical impoverishment.

In terms of financial risk protection, direct health payments by household fell gradually from 45% of total health spending in 1994 to 35% before UHC achievement, and <20% after the UCS 30 Baht (US$0.9) copayment was abandoned in 2006, and to <15% in 2010. Incidence of catastrophic health expenditure, measured by out-of-pocket payment >10% of total household spending, decreased substantially from 6% in 1996 to <3%–4% in the first decade of UHC [1]. There was a marked drop in the number of households impoverished by health payment after UHC was achieved (Figure 1).

**Figure 2:**
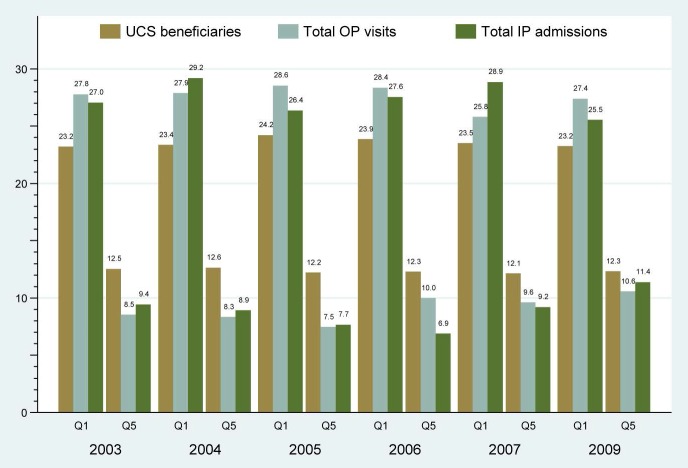
Health care utilization by the poorest and richest beneficiaries.

As a measure of service utilization, annual per capita outpatient (OP) visits and inpatient (IP) admissions have increased from 2.41 and 0.067, respectively, in 2003 to 3.22 and 0.112 in 2009, and to 3.64 and 0.119 in 2011. OP visits among the poorest quintile of UCS beneficiaries were disproportionately higher (26%–28%) than the richest counterpart (8%–10%) [5]. Similarly, IP admissions were concentrated more among the poorest quintile of the population than the wealthiest quintile (Figure 2).

Despite the achievement of UHC, challenges remain. An increased burden from NCDs is evident. Series of the NHES showed increased prevalence of overweight and obesity and diabetes in both male and female adults over the last two decades (Text S1). The cost pressure to all schemes from an increased demand for long-term treatments prompts policy attention towards effective primary, secondary, and tertiary preventions of priority NCDs, which address the social determinants of unhealthy lifestyles.

## Conclusions and Recommendations

Locally initiated and financed UHC, which is independent from donor resources and agendas, ensures sustainability; continual improvement of M&E systems that are used for policy decisions in line with national interests are the main features of the Thai experience. Factors contributing to these features are institutional capacities to generate evidence and influence policies; M&E with effective feedback for adjustment; economic growth and improved fiscal space; political and financial commitments; implementation capacities and supply-side resilience to accommodate significant increases in service utilization.

The M&E data platforms should be sustained and strengthened. Although the HWS and SES are adequate in measuring financial risk protection and utilization, they cannot monitor effective coverage. Key bio-markers, such as blood sugar for diabetes and blood pressure for hypertension captured by the NHES, are needed. Self-reported unmet health care needs embedded into the HWS, although subject to differences in expectation, are useful and should be continued. Finally, while hospital waiting times for key interventions are recorded in hospital level log books, it is done with great variation in definition and measurement and should be standardized and scaled up nationally.

## Supporting Information

Text S1 **The full country case study for Thailand.**
(DOCX)

## Abbreviations

HWS: Health and Welfare Survey
M&E: monitoring and evaluation
NCD: non-communicable disease
NHES: National Health Examination Survey
SES: Socio-economic Survey
UCS: Universal Coverage Scheme
UHC: universal health coverage
