# Supplementary material for: Monitoring and Evaluating Progress towards Universal Health Coverage in Thailand
Source: PLoS Med. 2014 Sep 22;11(9):e1001726. doi: 10.1371/journal.pmed.1001726 (PMC4171094; doi:10.1371/journal.pmed.1001726)
Supplement: Text S1 — The full country case study for Thailand. (DOCX) [file pmed.1001726.s001.docx]

**Full Case Study: Monitoring and evaluating progress towards Universal Health Coverage in Thailand**

Viroj Tangcharoensathien ^1^, Supon Limwattananon^1,2^, Walaiporn Patcharanarumol^1^ and Jadej Thammatacharee ^3^

^1^ International Health Policy Program (IHPP), Ministry of Public Health, Thailand

^2^ Khon Kaen University, Thailand

^3^ National Health Security Office, Thailand

*Corresponding author: Viroj Tangcharoensathien

Email: viroj@ihpp.thaigov.net

**This paper is the full country case study to accompany the summary paper “Monitoring and evaluating progress towards Universal Health Coverage in Thailand” that is part of the Universal Health Coverage Collection. Not commissioned; externally reviewed.**

**Abstract:** By 2002, all Thai citizens were covered by a financial risk protection system with a set of comprehensive service package, resulting in low level of out of pocket payment, 14% of total health expenditure and high level of financial risk protection. This paper reviewed and analyzed how different data platforms and indicators essential for monitoring and evaluating (M&E) progress towards Universal Health Coverage (UHC) were developed.

At the 2001 inception of Universal Coverage Scheme (UCS), there was no explicit M&E blueprint; development of information systems and indicators had been incrementally built on the existing platforms in a pragmatic way. Computerized Civil Registration system covering almost all births (96.7%) and deaths (95.2%), member databases held by three insurance schemes, sharing and interoperability of these data bases not only contributed to accurate headcounts of births and deaths but was used to ensure citizen entitlement to healthcare.

The existing national representative household surveys conducted regularly by National Statistical Office, notably Socio-Economic Survey, Health and Welfare Survey and the National Health Account contributed to the assessment of level and distribution of utilization, benefit incidence and financial risk protection measured by incidence of catastrophic health expenditure and health impoverishment. National Health Examination Survey contributed to measurement of effective coverage of key non-communicable diseases. The routine administrative data and various diseases registries were established in responses to program management requirements.

Locally initiated, domestically financed and independent from donors' agendas and resources ensured sustainability, continued improvement and use for policy decision in line with national interests.

**Summary Points:**

1. Two strands health systems development were applied: expansions of functioning district health systems in 1970s-1980s and financial risk protection extension, targeting different groups of population since 1975; these are platforms facilitating smooth implementation of Universal Coverage Scheme (UCS) in 2002 with a favorable outcomes.

2. Tax-financed UCS is progressive, a close-end provider payment is financially feasible to offer a comprehensive benefit package with very minimum copayment, resulting in low level of household direct payment, high level of financial risk protection, minimum incidence of catastrophic health spending and impoverishment.

3. At UCS inception, there were no explicit monitoring and evaluation (M&E) blueprints. Information systems for M&E and indicators were incrementally built on the existing platforms. Civil Registration facilitates accurate population coverage and ensured citizen entitlement to health services. Health and Welfare Survey and Socio-economic Survey are backbones for the assessment of level and distribution of health utilization, benefit incidence and financial risk protection. Series of National Health Examination Survey (NHES), locally initiated and financed, contributes to measurement of effective coverage of key non-communicable diseases.

4. Locally initiated and domestic financed UHC, independent from donor resources and agenda ensured sustainability; continue improvement of M&E systems for policy decision are main features of Thai experiences. Factors contribute to these features are institutional capacities to generate evidence and influence policies, M&E capacities and effective feedback; economic growth and improved fiscal space, political and financial commitments; implementation capacities and supply side resilience to accommodate significant increases in service utilization.

5. We suggest sustaining and strengthening M&E platform, more frequent NHES for monitoring effective coverage of key NCD; continue embedding self-reported unmet healthcare needs in regular surveys by National Statistical Office and establishing hospital waiting time of key interventions.

**1. Background**

By 2002, all Thai citizens were covered by one of the following three financial risk protection schemes: Civil Servant Medical Benefit Scheme (CSMBS) for government employees and dependants, Social Health Insurance (SHI) for private employees and Universal Coverage Scheme (UCS) for the remaining Thai citizens not covered by two other schemes. Stateless and displaced persons either pay out of pocket payment, or are exempted by public providers until recently some 0.4 million was financed by general tax. Very few registered migrants were covered by premium-financed health insurance scheme managed by Ministry of Public Health (MOPH); while the majority non-registered migrants pay out of pocket for health services **[1]**.

Table S1 offers an overview of three public insurance schemes; historical precedence and territorial ownership of CSMBS and SHI opposed a single fund for the whole population during the 2002 reform; hence a new UCS emerged. While keeping an existence of the three schemes, the current reform direction, though not so successful, was to harmonize benefit packages, the way and magnitude providers were paid. The most outstanding problem was the unsuccessful reform of fee-for-service payment for outpatient care in CSMBS which resulted in an inefficiency and rapid cost escalation. See Jongudomsuk et al **[2]** for political dimensions and failure to reform**.** Box S1 describes key feature of the provider payment methods.

|  | **CSMBS** | **SHI** | **UCS** |
| --- | --- | --- | --- |
| 1. Establish since | 1980 | 1990 | 2002 |
| 1. Responsible agency | Ministry of Finance, Comptroller General Dept. | Ministry of Labour, Social Security Office | National Health Security Office |
| 1. Legal status | Royal Decree | 1990 Social Security Act | 2002 National Health Security Act |
| 1. Population coverage, % of total population | Gov employee, pensioners + dependants, 9% | Private sector employees, no dependents, 15% | Remaining population, 75% |
| 1. Finance source | General tax, through annual budget bill | Tripartite, 4.5% payroll, 1.5% each | General tax, through annual budget bill |
| 1. Provider payment | Fee for services for outpatient care, DRG for inpatient care with X bands of cost weight in favour of teaching and tertiary hospitals | Inclusive capitation for outpatient and inpatient care, except IP where cost weight higher than 2 is paid on a DRG system | Capitation for outpatient care, Global Budget and DRG for inpatient care, single base rate to all providers |

**Table S1:** Key characteristics across three public health insurance schemes, 2013

Data source: Authors’ synthesis

| **Box S1: Key feature on provider payment mechanisms**  Closed end provider payment using capitation to pay inclusively outpatient and inpatient care adopted by SHI since 1991 was the predecessor of the UCS in 2002 to adopt capitation for paying outpatient services, and global budget and Diagnostic Related Group (DRG) for paying inpatient services.  DRG was adopted by CSMBS to pay inpatient care since 2008 replacing conventional fee for service without control. As all three Schemes applied closed-end payment (except CSMBS fee for service for outpatient) contributed to cost containment, value for money and overall systems efficiency. A few low-and middle income countries had such close end payment applied for the whole countries and suffered from cost escalation as mostly applied fee for service such as China **[3,4]**. |
| --- |

In this paper, we address progress towards Universal Health Coverage (UHC) based on the WHO three-dimensional framework **[5]**. We focus around an advent of the UCS as the landmark of UHC achievement when the whole population is covered, the other two dimensions, service coverage and financial risk protection were gradually expanded when there is more evidence such as cost effectiveness of new interventions and improved fiscal space for health.

Evidence demonstrates favourable UHC outcomes **[6,7]**: improved and equitable access to health services and low level of unmet health needs **[8]**, high level of financial risk protection, as measured by low incidence of catastrophic health expenditure **[9]** and reduced number of non-poor households becoming poor after health payment given the comprehensive benefit package provided free of charge even the costly interventions.

This paper reviewed population health achievements and health systems development over three decades (1970 to 2000) paving a platform for an implementation of the UCS--the largest public scheme covering 75% of population in 2001-02; analyzed how different data platforms and indicators essential for monitoring and evaluation (M&E) progresses of UHC were developed and used for policy decisions; and synthesized a few key indicators on progress towards UHC goals.

**2. Universal health coverage: the policy context**

***Health achievements in the last three decades***

Thailand ranked the first among 80 countries with the highest average annual reduction (8.5%) in child mortality between 1990 and 2006 **[10]**. As a result of the high coverage of MCH services, for example, 99% skilled birth attendants since 2000, 79.6% contraceptive prevalence rate (all methods) among women 15-49 years in 2009 **[11]**; and a reduction in total fertility rate from 5.0 in 1970s to 1.6 in 2000s. High coverage resulted in narrow urban-rural and rich-poor gaps of service coverage **[12]**. These basic but essential services were provided mainly by the district health system (DHS), including sub-district health centres and district hospitals which are geographically accessible by the rural poor people. DHS is the strategic hub, translating policy statements into equitable outcome, as it is geographically accessible and commonly used by the rural population who are mostly poorer.

Life expectancy increased from 63.8 years in 1975 to 77.6 years in 2005; female outpaced male. A decade, 1995-2005, stagnation in the life expectancy at 70 years was observed among men due to AIDS epidemics which claimed prime adults **[13]**. This was confirmed by the 1999 and 2004 studies on burden of diseases that HIV was the leading cause of Disability-adjusted Life Year loss **[14,15]**.

While infant and child mortality demonstrated a consistent and continual improvement **[16]**; reduction in adult mortality showed mixed results. The probability that an individual who has just turned 15 years will die before reaching 60 years old slightly increased in the Thai men from 198 per 1,000 in 1990 to 200 in 2010 due to AIDS epidemics and lack of effective control intervention for road traffic injuries and fatalities affecting prime adults **[17]**.

Thailand marked a good record in health achievement **[10]** through comprehensive primary health care. Since the early 2000s, all health MDGs have already achieved. Owing to sustained political commitments. HIV was reversed from generalized to concentrate epidemic since 1991; annual new infections decreased from 143,000 in 1991 to 19,000 in 2003 **[18]**. The universal Anti-Retroviral Therapy (ART) launched in 2003 significantly reduced mortality. When national goals were achieved, specific sub-national targets were set for disadvantage areas such as Northern mountainous and Southern Muslim provinces **[19]**.

***Three decades of health systems development: key platforms for the UCS implementation***

Health systems development since 1970s contributing to effective implementation of UCS in 2002 was categorized into two broad strands, see Box S2.

| **Box S2:** Two strands of health systems development  ***Minimize physical barriers***  Expansion of health services focusing at district health systems was the national agenda since the fourth National Socio-Economic Development Plan (1977-1981); it was an integral part of rural development, poverty reduction and national security in the context of anti-communism in Asia and "domino theory" put forth by American and Thai government allies, especially after the conclusion of Vietnam war in 1975. It aimed a full coverage of district hospitals in all districts and a health centre for every sub-district covering a typical five though population in its catchment area. By the end of sixth National Socio-Economic Development Plan, in 1991, all sub-districts and districts were fully covered by health centre and district hospitals, serving as "close-to-client", better accessed health service throughout the country.  Primary health care can only functioning when there are adequate number of committed health workforce, especially in hard to reach districts. Production and rural deployment of health personnel through government bonding of rural district health services for all medical graduates was launched since 1973 and later extended to nurses, pharmacists and dentists. Integrating education strategies with rural retention objectives such as recruitment of rural students for medical and nursing education, trained them in provincial medical and nursing schools and their hometown placement upon graduation had shown positive outcome in term of better rural attitudes, intention to serve longer period in rural areas **[20]**. Undeniably, the MOPH high level production capacity of medical and nursing and other health-related personnel contributed significantly to the functioning of rural health services.  ***Minimize financial barriers***  The extension of financial risk protection through piecemeal targeting approach, addressing the poor and vulnerable covered by tax-financed Medical Welfare Scheme in 1975 when the GNI per capita was as low as US$ 380; it gradually extended to government employee covered by tax-financed Civil Servant Medical Benefit Scheme in 1980, private sector employee covered by payroll-tax financed scheme in 1991; and informal sector covered by Voluntary Health Card Scheme as contributory since 1983 and became a public subsidized voluntary health insurance when half of the 1,000 Baht premium for the whole family was subsidized by the government in 1995.  Gradual expansion of individual schemes was applied; in 2001, 30% of the people were still uninsured. When the window of opportunities opened for UHC as a political manifesto on the 6 January 2001 general election, the Government led by Prime Minister Thaksin Shinawatra adopted UHC policies. UHC is a political decision, while policy formulation and systems designs are guided by evidence. Pilots in six provinces in April 2001 was extended to 21 provinces in June 2001 and scaled up to the whole country by April 2002 **[21]**. A National Health Security Act was endorsed by the Parliament in November 2002. |
| --- |

Figure S3 depicts the advents of different financial risk protection schemes **[6]**. By 2002 the whole population was covered by one of the three financial risk protection schemes when Thailand’s gross national income (GNI) per capita was merely US$ 1,900, a lower-middle income status. It took 27 years since the medical welfare scheme for the poor was launched in 1975. It hints that a country does not have to be rich to reach the UHC. Though UCS benefit package at the 2002 launch was comprehensive; adding Anti-retroviral therapy in 2003, Renal Replacement Therapy in 2006 and other new interventions guided by evidence such as cost effectiveness as well as budget impact analysis **[2]** further deepened financial risk protection **[7,21]**.


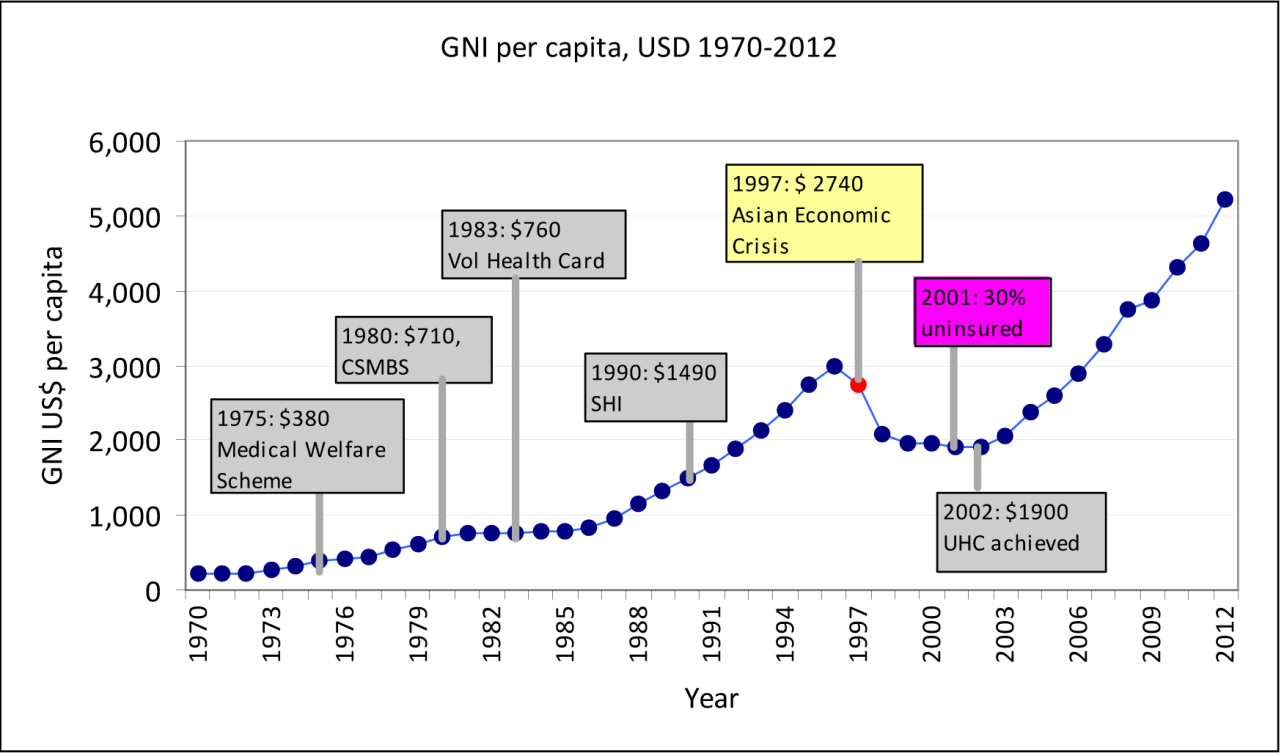


**Figure S3:** UHC trajectory and GNI per capita, USD 1970-2012

Data Source: [6]

The pro-poor outcome as indicated by healthcare utilization and government budget subsidies disproportionately benefited the poor **[22]** was attributed to government capacity to deliver effective services by DHS with its extensive geographical coverage. Other enabling factors are charismatic leadership of Ministry of Public Health (MOPH) in the past, successive nine five-year National Health Plans ensuring continuity of the health systems development, a number of idols transcending the intrinsic factors to younger generation of health care providers who are implementers in rural areas **[23]**.

**3. Monitoring and evaluation for UHC**

***Ministry of Public Health M&E framework***

Health sector M&E frameworks have gone through series of evolution; key data platforms have been developed in response to these frameworks. When health facilities reports were incomplete e.g. not able to estimate the denominators and exclude private sector; household surveys were used to estimate coverage, such as EPI coverage survey and the MOPH surveys of health infrastructure and workforce.

Owing to advancement of information technology and internet coverage to all health facilities, the current M&E framework relied solely on routine administrative reporting. However, it covers only MOPH health facilities excluding non-MOPH public and private health facilities. In the fiscal year 2013, 81 key performance indicators were required by MOPH **[24]**, mostly service coverage for NCDs such as diabetes, hypertension for which each province had to report their achievement. This system was recently established; reporting was less than 75%. With an unsettled denominator, either all facility users or catchment population, it is difficult to document reliable coverage rate. The system has yet to extend coverage to non-MOPH public and private sectors, as they contribute to more than 26% and 24% of total outpatient and inpatient caseloads **[25]**. Besides, it did not cover other social determinants of health which lie outside health sector such as tobacco, alcohol and physical in-activity.

***UHC M&E framework: data platform and key indicators***

Table S2 (end of the paper) compiles data platforms evolved in responses to indicators; these are classified into four dimensions: input, output, outcome and impact of UHC.

***Inputs***

National Health Account (NHA) was locally innovated in 1994 **[26]**, sustained and institutionalized; The International Health Policy Program (IHPP) was designated as the NHA national focal point. In response to UNGASS on HIV/AIDS, the National AIDS Spending Account has been developed and sustained since early 2000s. The NHA working group was successful in improving SES questionnaire capturing the rare events of inpatient care and expenditure, by asking these events in the last 12-month, instead of last month recall period for both outpatient and inpatient expenditures, and linked expenditure with services used. The long series of NHA since 1994 was the useful basis for a twenty-year projection of total spending as percent of GDP **[27,28]**. The Socio-economic Survey (SES) contributed to the assessment of financial risk protection, see Box S3.

| **Box S3**: Key data sources for monitoring progresses towards UHC  Health and Welfare Survey and Socio Economic Survey conducted regularly by NSO are the two important data source for monitoring equity in health service utilization and financial risk protection.  **Health and Welfare Survey**  First conducted by NSO in 1974, then every five year. In response to UCS launched in 2002, IHPP requested for an annual survey between 2003-2007, now every two years. The 2013 survey conducted in March 2013 was the 18th round. It is a national representative health interview survey, using multi-stage random sampling technique, sample size was large: 27,960 households in 2013.  Core module includes questions on insurance coverage, illness in the last month, hospitalization and dental services in the last 12 months, choices of health service used for outpatient an inpatient, unmet healthcare needs, out of pocket payment. Special modules were inserted in some rounds e.g. use of tobacco and alcohol, physical activities, screening of breast and cervical cancers.  The survey contributes to equity monitoring of healthcare use by wealth quintiles, urban-rural, province, region and gender. It contributes to the assessment of benefit incidence.  **Socio Economic Survey**  First conducted by NSO in 1957 called Household Expenditure Survey; it was renamed as SES in 1957 and conducted every five years. In responses to policy use for poverty reduction and income distribution, it was conducted more frequently every two years since 1987, and annual since 2006, the survey in 2013 was the 25th round. It is a national representative household interview survey, using multi-stage random sampling, total samples were 52,000 households in 2012, interviews were conducted throughout the years.  Content covers household income by all sources, expenditure on all items including health, tobacco, alcohol; debts and loans from all sources. Use to construct National Health Account (household health payment), assessment of incidence of catastrophic health expenditure.  Out of pocket payment generated from SES contributes to the estimate of the incidence of catastrophic health expenditure, and health impoverishment; it can be stratefied by wealth quintiles, urban-rural, province, region and gender It contributes to the assessment of benefit incidence. It contributed to the construction of National Health Account on household health payment as well as the construction of national poverty line by geographical region and urban and rural; additional inputs for the assessment of health impoverishment. |
| --- |

Administrative records on number of health workforce in public and private sectors were weak, especially in the context of full- and part- time arrangement; the annual Health Resource Survey conducted by MOPH captured data in the public and private (though coverage is low) sectors contributed to monitoring of health workforce and infrastructure distribution and density, reflecting inequity across regions.

***Outputs***

Though the Civil Registration was mandatory by law since 1909, for households to register all births and deaths; rapid progress was observed since 1982 when the unique citizen identification number assigned to all citizen since birth, was initiated and gradually transformed to computerized systems. All births and deaths were mandated by law to register within 15 days and 24 hours respectively. The daily updated Civil Registration was shared with the National Health Security Office (NHSO), representing three insurance schemes, to update their insurance registries.

The level and distribution of utilization can only be assessed by the NSO’s Health and Welfare Survey (HWS), see Box S3. Other specific surveys such as Elderly, Disable, Reproductive Health, and Multi-Indicator Cluster Survey (MICS) are useful evidence on specific target groups. The annual per capita use rate generated by HWS was applied for calculation of per capita budget requirement for UCS between 2002 and 2006. NHSO administrative data on utilization of inpatient and outpatient, completely developed in 2006 and 2010 replaced the HWS. However still HWS contributed to equity monitoring.

Healthcare Accreditation Institute was the key player in strengthening quality of health services provided by public and private sectors. The Institute assessed health facilities (health centres and hospitals) based on a standard protocol for accreditation and re-assessed for re-accreditation every three years **[29]**. Accreditation status was used for additional pay by the NHSO to incentivize quality improvement.

***Outcome***

Service coverage indicators captured by health facility data are numerous, e.g. quality antenatal care of four visits, skilled birth attendance, child immunization, family planning, Prevention of Maternal to Child HIV Transmission (PMTCT) and ART. When the service coverage such as PMTCT, ART and renal dialysis was used for annual budget approval; the quality, coverage and accuracy of the data were significantly improved.

The outcome of prevention interventions, such as smoke free environment (measured by second hand smoker), smoking among pregnant women, smoker quit rate, use of alcohol among under age (below 20 years old according to the 2008 Alcohol Control Act) were captured by NSO household surveys. These surveys were closely coordinated between NSO and users **[30,31]**. The National Health Examination Survey (NHES) based on clinical examination and laboratory tests produced more reliable NCD prevalence.

The question if the government’s UCS budget benefited the rich or the poor--a benefit incidence analysis was answered by the existing household surveys, notably SES and HWS. The pro-poor outcome of benefit incidence was reported to general public and the government; fostering adequate budget allocation to the UCS.

***Impact***

The first National Health Examination Survey (NHES) was initiated in 1991, the subsequent waves 2, 3 and 4 were conducted in 1997, 2004 and 2009 respectively **[32,33,34,35]**; the ongoing fifth wave was in 2013. Sample household members were interviewed, their blood pressure was measured, anthropometry was conducted and blood samples were drawn for tests. Effective coverage generated by NHES was measured by the proportion of hypertension that were diagnosed or screened and were on anti-hypertensive treatment and were well controlled. Low effective coverage for diabetes, hypertension and hyperlipidemia has led to active screening and treatment campaigns recently.

A enquiry on health systems responsiveness with an application of the WHO concepts was recently initiated as a special module of HWS in 2013. To counteract the potential downside of capitation payment, in particular poor quality and under provision of care, annual consumer and provider satisfaction survey has been assessed by an independent poll commissioned by NHSO since 2003. At local level, hospitals monitored satisfaction of their clients on a regular basis and used results for service improvement.

**4. Progress towards UHC in Thailand**

The trends of selected indicators on progress towards the UHC goals were synthesized in this section.

***Inputs***

In 1994, 45% of the total national health expenditure was paid by households, whereby the government shared a similar portion (Figure S4). The direct health payment fell gradually, to approximately 35% the period just right before the UHC achievement, then substantially dropped to less than 20% after the 30-Baht copay requirement from the UCS members was fully abandoned in 2006; see political dimensions of termination copayment **[6]**. The health expenditure shared by household payment further reduced to less than 15% in 2010, much lower than the average of Organization for Economic Co-operation and Development (OECD) 17.9% in 2010 **[36]**.


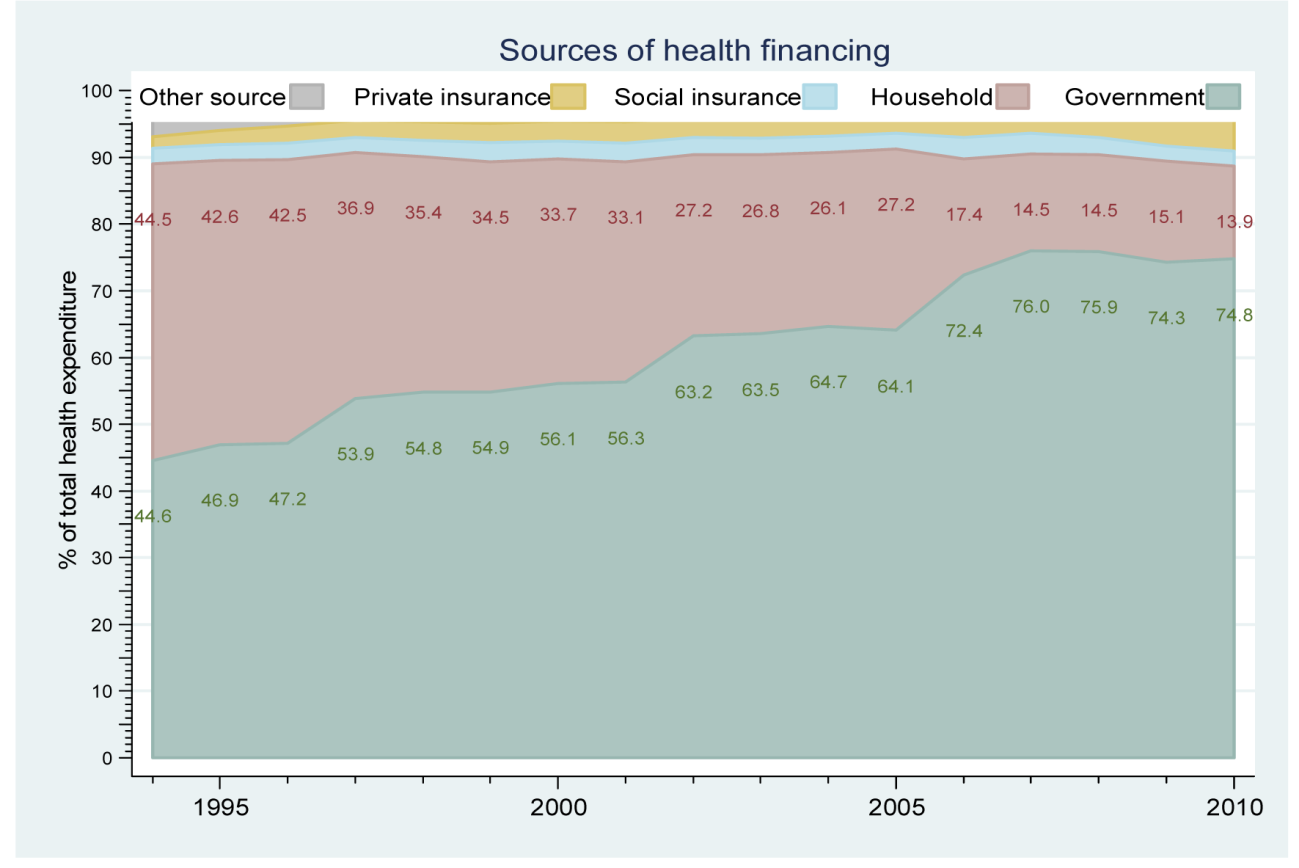


**Figure S4:** Sources of financing health care, 1994-2010

Data Source: National Health Account, various years

Almost three decades before achieving the UHC, there was a rapid geographical expansion of public hospitals at the district level (Figure S5). At present, every district in Thailand had at least one district hospital. A typical district hospital with 10-90 beds and approximately 3-10 full time medical doctors and 20-200 registered nurses serve as the main contracting unit for the UCS.


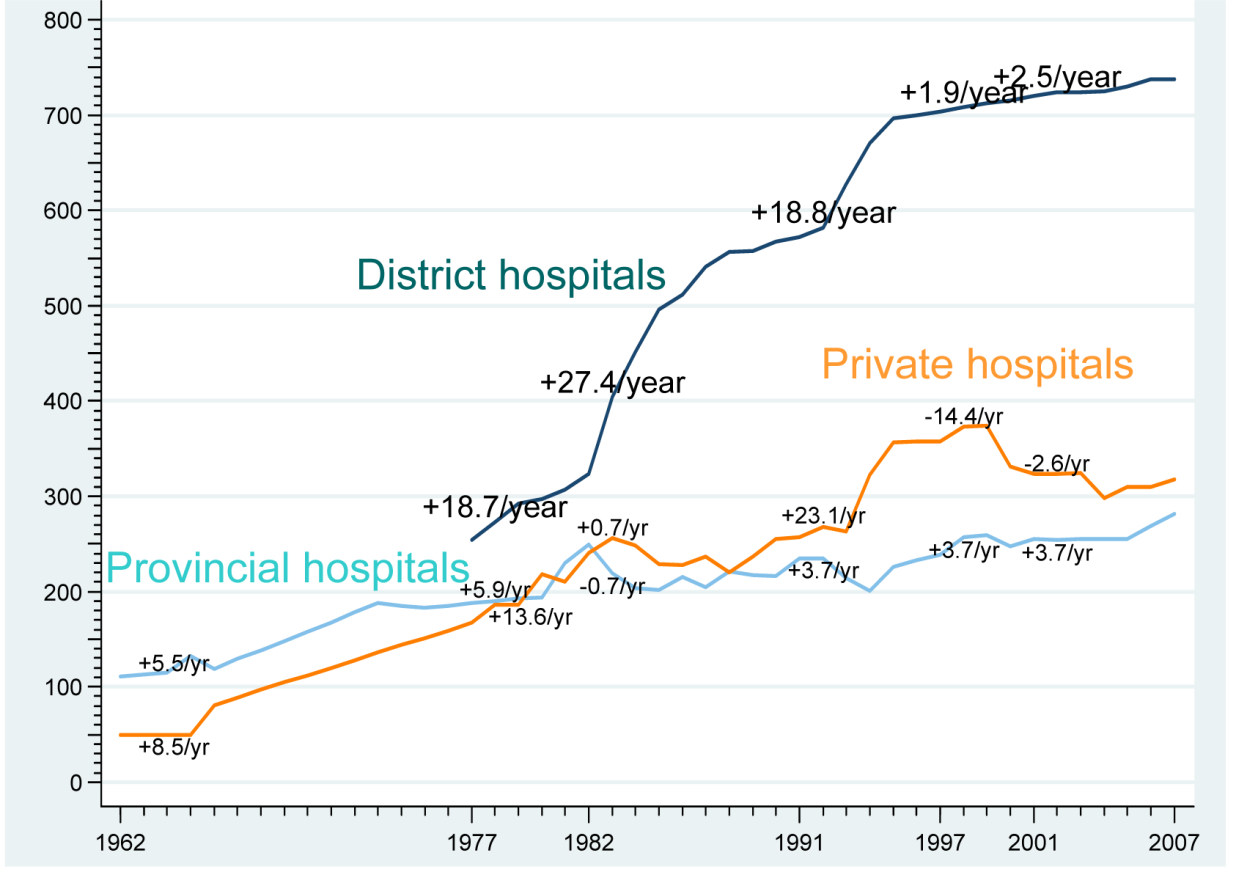


**Figure S5:** Expansion of public and private hospitals 1962-2007

Data Source: Authors’ analysis

***Outputs***

An overall utilization of health services continued to grow over the first decade of the UHC achievement despite relatively stable number of the beneficiaries. The annual per capita OP visits and IP admissions increased from 2.41 and 0.067 respectively in 2003 to 3.22 and 0.112 in 2009 and predicted to increase to 3.64 and 0.119 in 2011 (Figure S6).

**A**


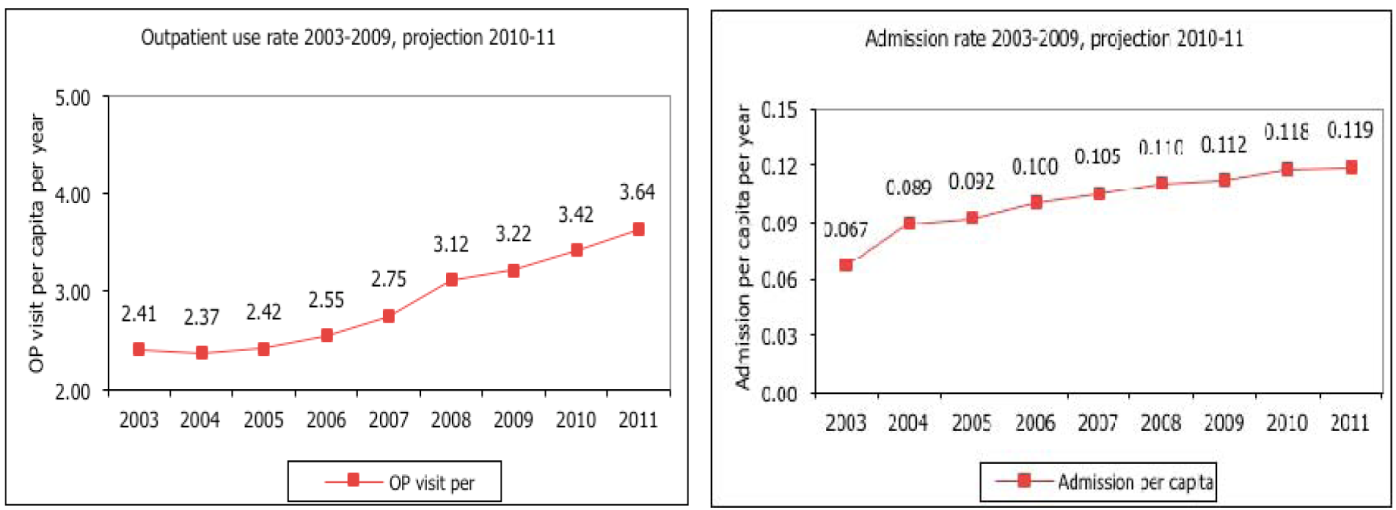


**B**


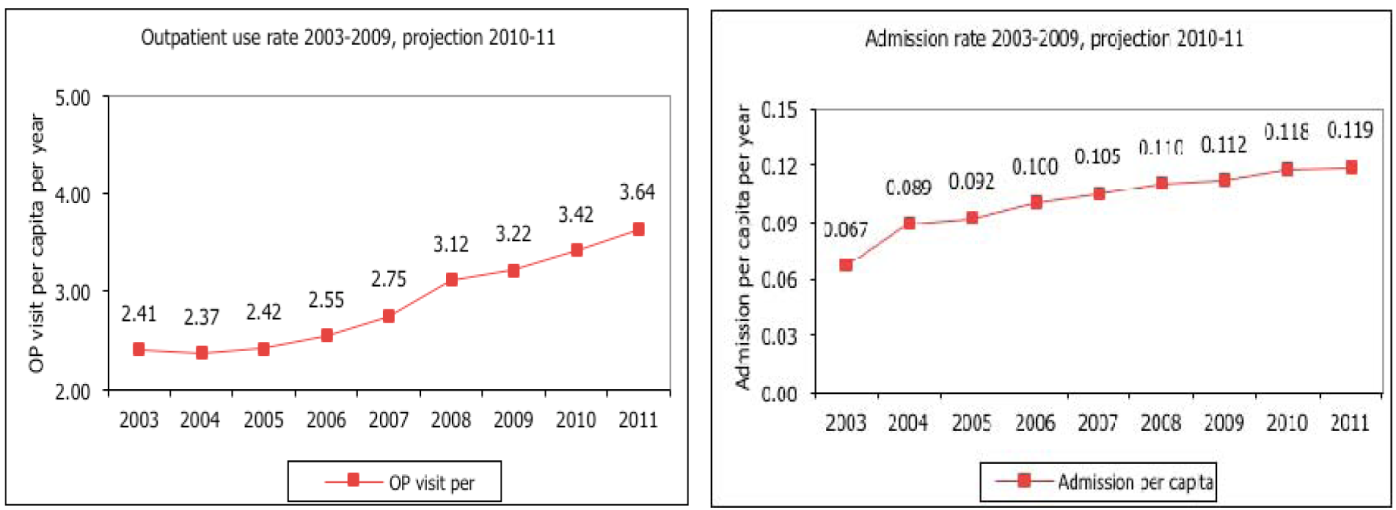


**Figure S6:** Increased OP visits and IP admissions, 2003-2011. **A**: Outpatient use rate 2003-2009, projection 2010-11. **B**: Admission rate 2003-2009, projection 2010-2011.

Data Source: [6]

Across quintiles of the household’s asset indices, the poorest quintile of UCS beneficiaries used the OP service disproportionately higher (26-28% versus 8-10%) than the richest counterpart during the first decade UHC achievement (Figure S2). To a similar degree, the IP admissions concentrated more among the poor than the rich over the same period. The pro-poor utilization outcome was the results of NHSO contracting services with the better accessible district health system.


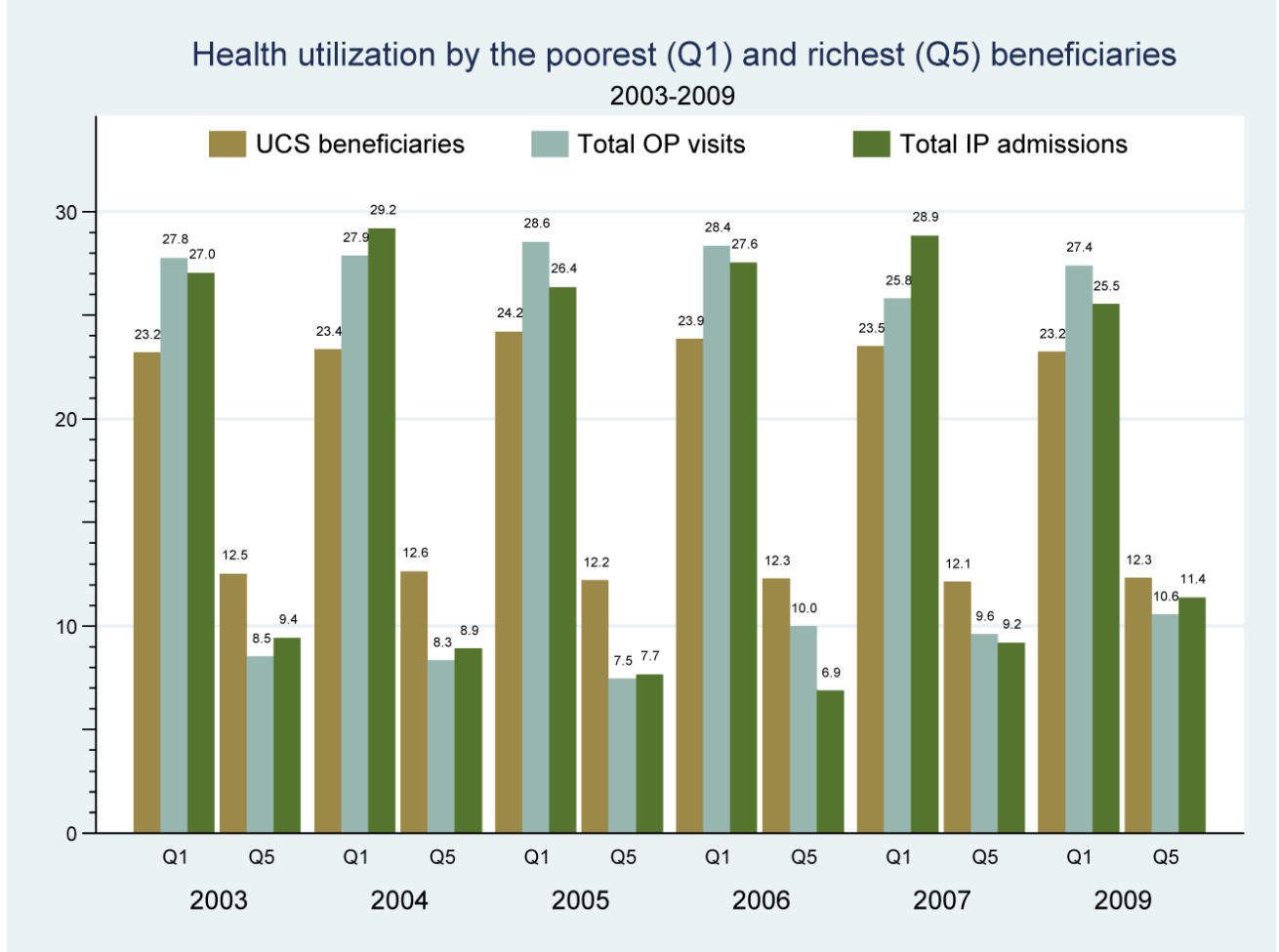


**Figure S2:** Pro-poor health service utilization, OP visits and IP admissions, 2003-2009

Data Source: [22]

There are four steps of progresses towards hospital accreditation. Step zero means hospitals are starting the processes or the accreditation status was expired. Step one means hospital had installed systems for risk preventions. Step two means hospital quality assurance and quality improvement was in place. Step three means hospitals were accredited or had been re-accredited after three years by the Healthcare Accreditation Institute. Figure S7 showed rapid transformation from Step 1 to 2 after 2007 quality incentives offered by NHSO. Note the increase of Step 0 from 1% in 2011 to 14% in 2012 was the effect of hospitals having their accreditation status expired after 3 years and not yet re-accredited.


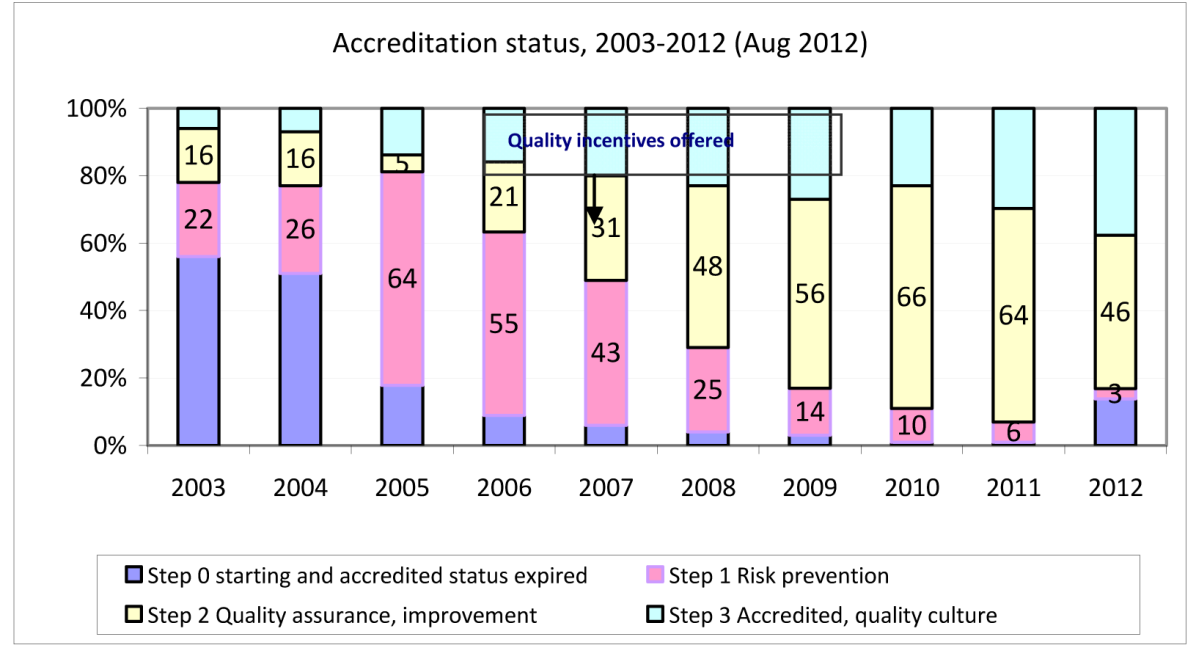


**Figure S7:** Hospital accreditation status 2003-2012 and quality incentives offered by NHSO in 2007

Data Source: NHSO’s data

***Outcome***

Reduction in financial risk and prevention of health impoverishment was a success story. Incidence of catastrophic health expenditure, defined as the Out-of-Pocket (OOP) payment above 10% of household total expenditure reduced substantially from 6% on average in 1996 to less than 3-4% during the first decade of UHC (Figure S8), if cut-off by the threshold of more than 40% of non-food expenditure, the incidence was much lower. A decreasing trend was apparent in both the richest and poorest quintiles.


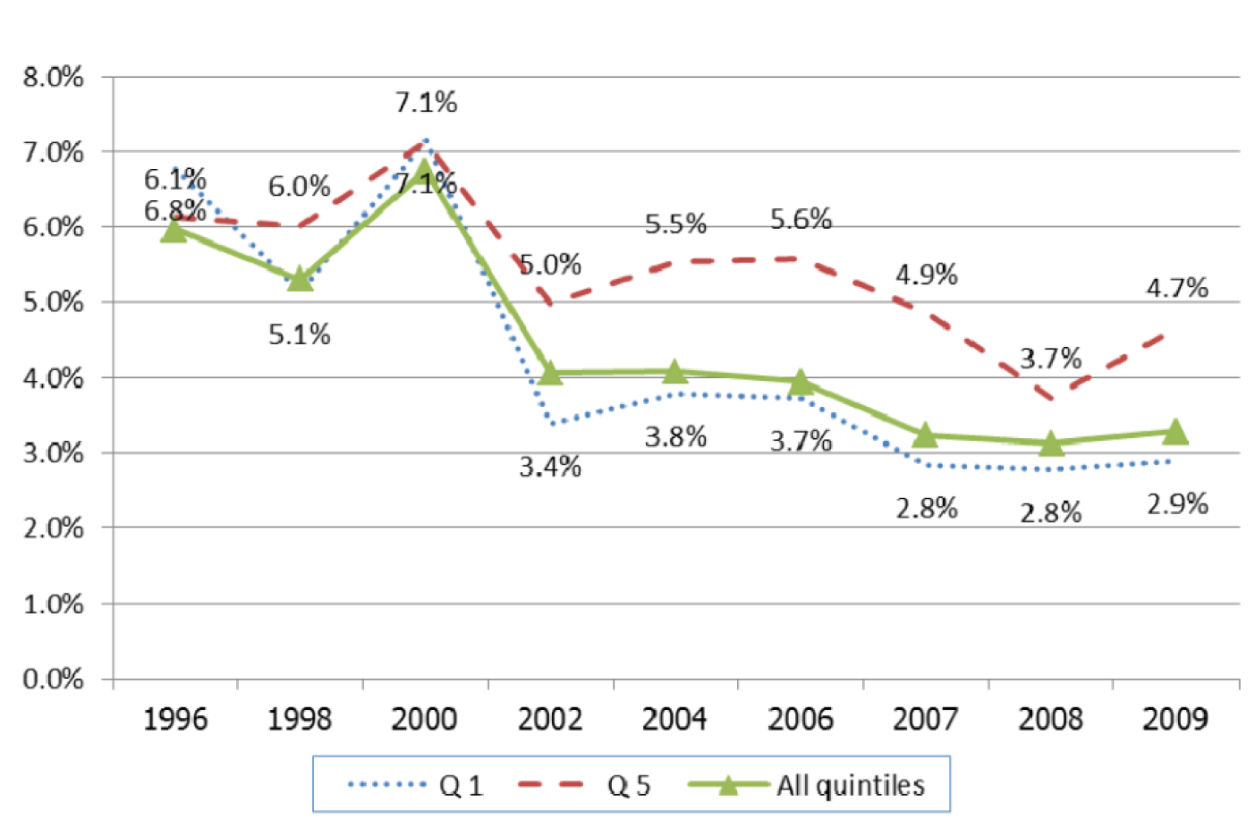


**Figure S8:** Incidence of catastrophic health expenditure (defined as OOP payment above 10% of total expenditure), poorest and richest quintiles 1996-2009

Data Source: [6] and [7]

There was a marked drop in the number of households impoverished by health payment after UHC was achieved (Figure S1). The dotted lines, representing the counterfactual scenario had there been no UCS policy between 2002 and 2009, was a projected trend line from 1996-2002. The difference between the counterfactual (dotted line) and the actual observed numbers of impoverished households (solid line) was the net number of non-poor household prevented from being poor due to health payment; the direct effects of UCS.

Note that SES and sub-national poverty line facilitate the construction of sub-national indicators on the incidence of catastrophic health spending and health impoverishment, but not possible for other breakdown such as insurance type, as there is no poverty line for members in each insurance scheme.


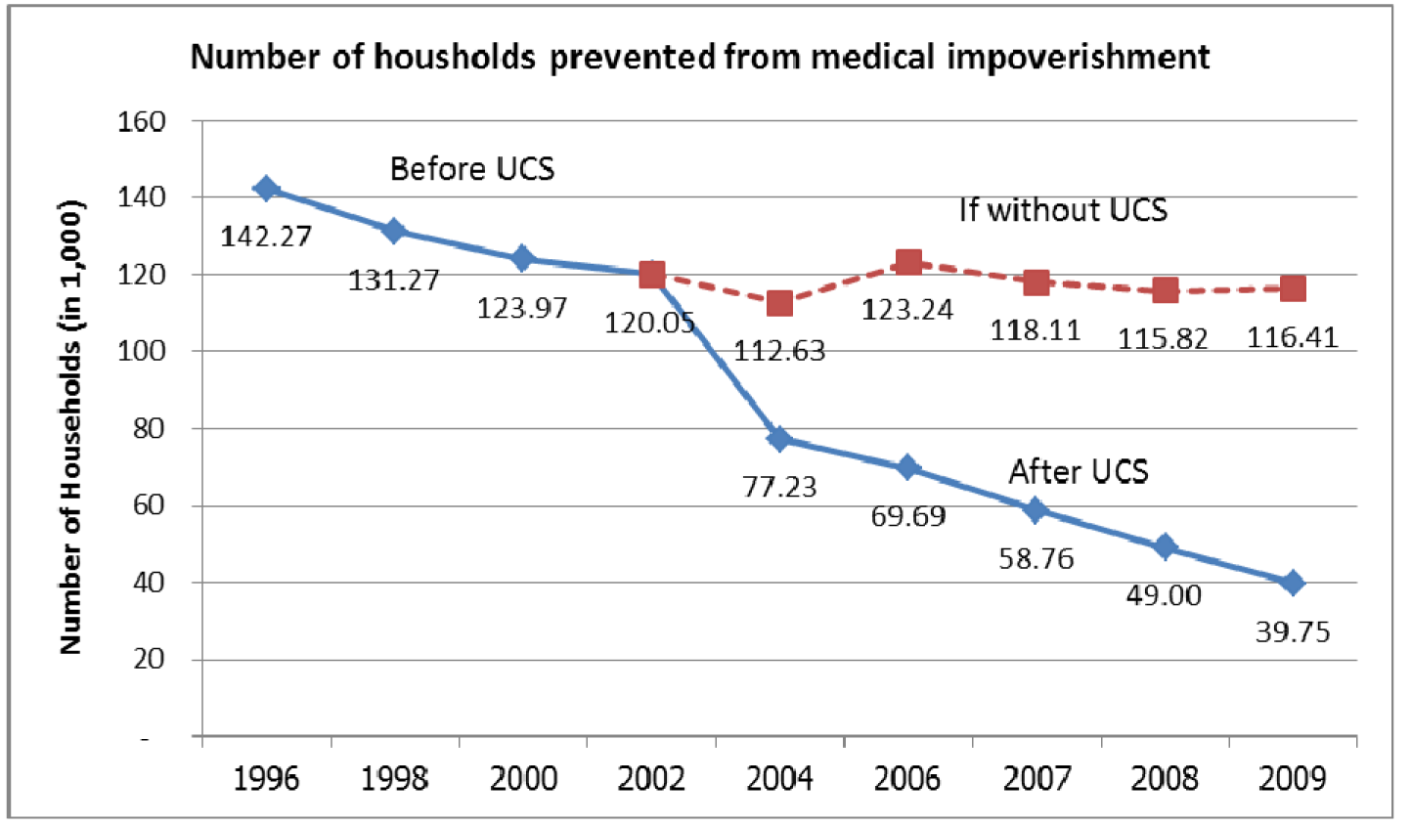


**Figure S1:** Reduced health impoverishment

Data Source: [6]

The pro-poor utilization of health services and the reduced OOP payment for health, especially among the poor contributed to the pro-poor government subsidy for health (Figure S9).


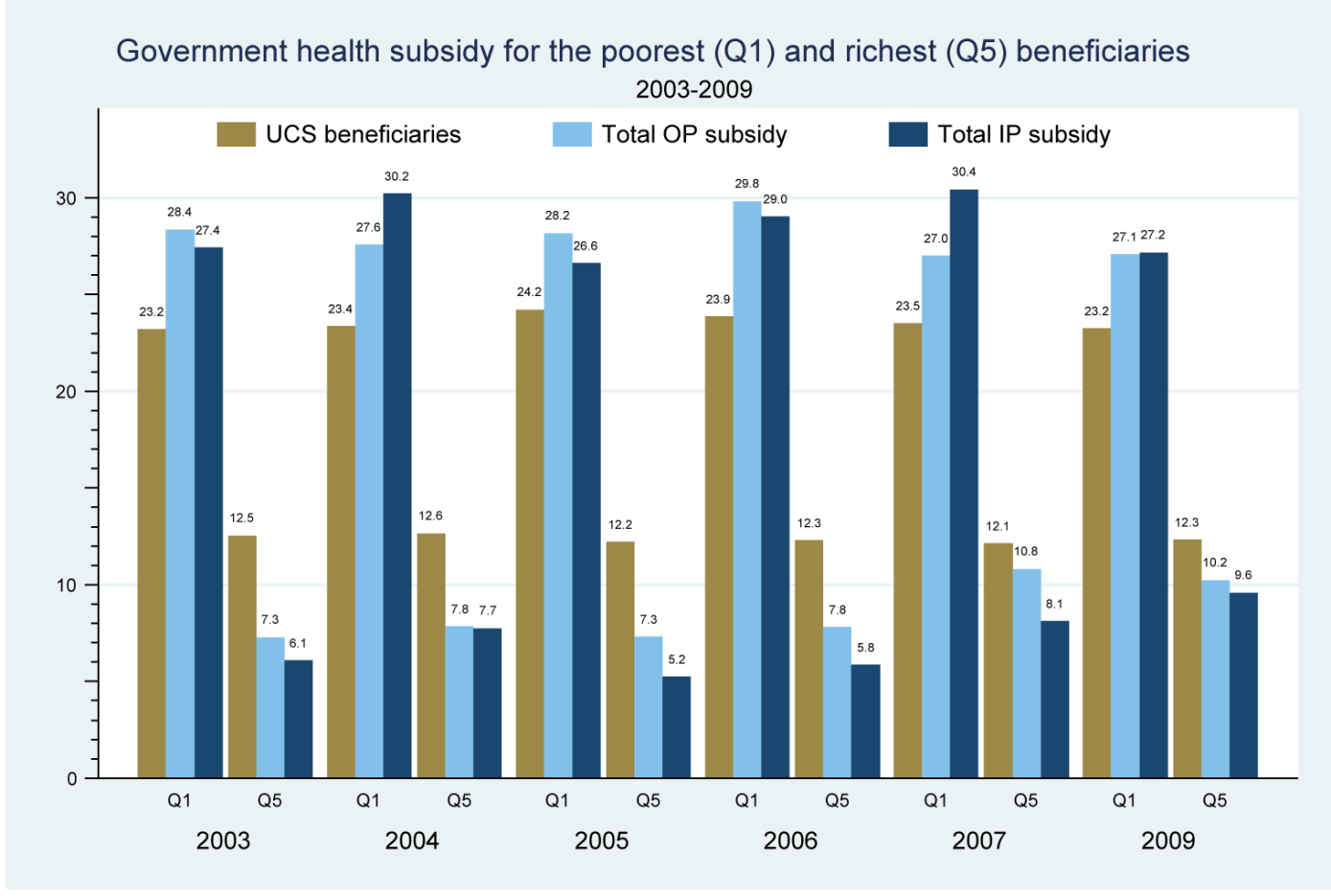


**Figure S9:** Pro-poor government subsidy (2003-2009)

Data Source: [22]

***Impact***

Increased burden from chronic non-communicable diseases was evident. Data from four rounds of NHES showed increased prevalence of overweight and obesity and diabetes in both male and female adult populations over the last two decades (Figure S10). Cost pressure from increased demand for long term treatment prompted policies towards effective primary prevention, early screening and treatment, prevention of complication such as end stage renal disease from diabetic and hypertension and diabetic retinopathy. Gruber et al **[37]** reports a sharp equalization and a large aggregate reduction in infant mortality of 13-30% between 2000 and 2002, probably attributable to UCS.

**A**


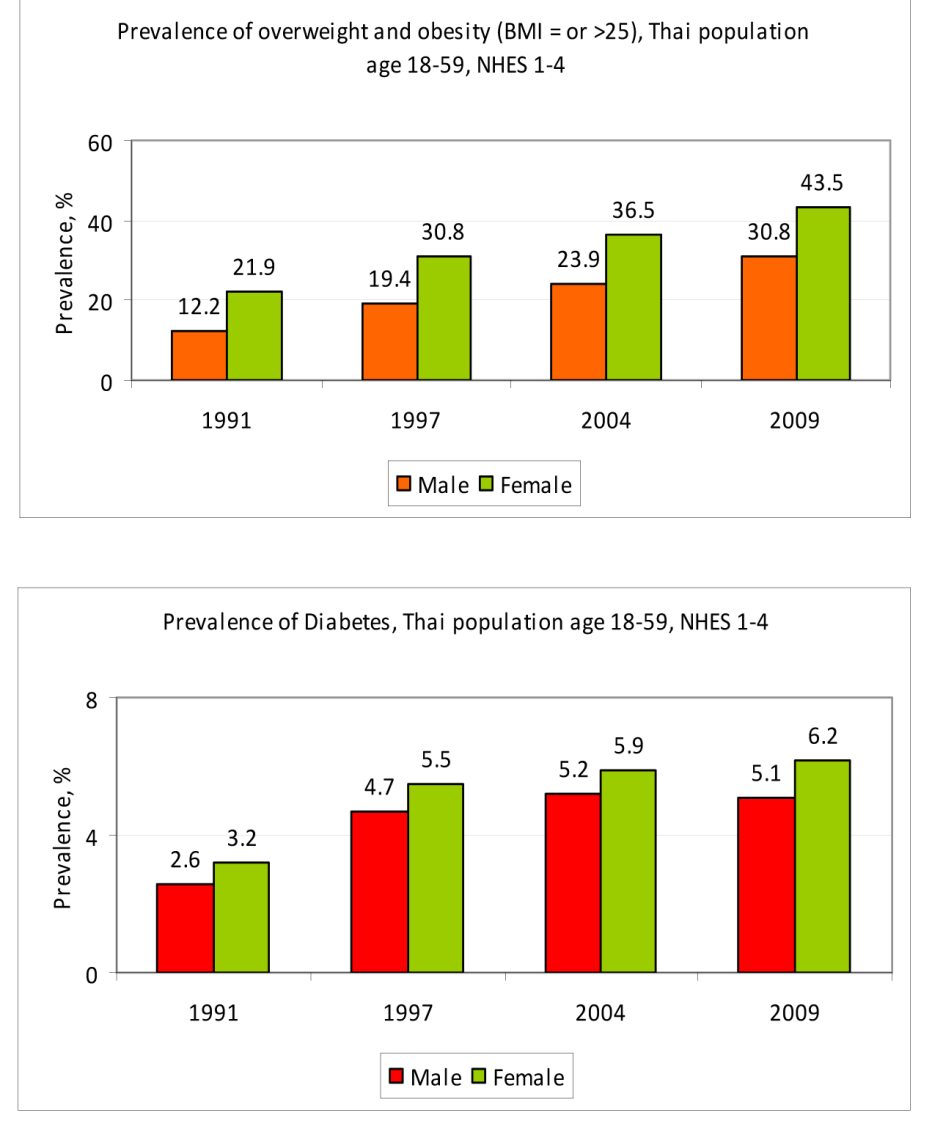


**B**


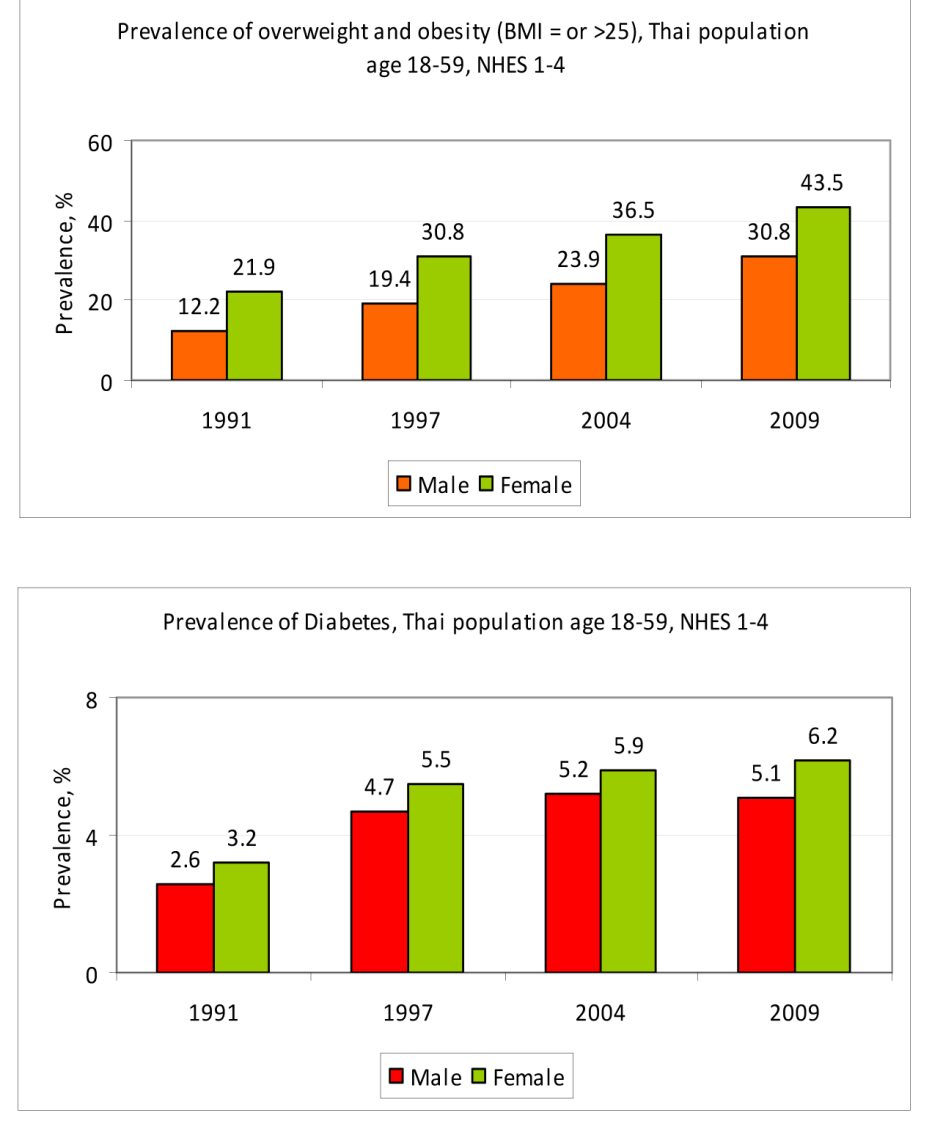


**Figure S10:** Prevalence of overweight and obesity, and Diabetes between 1991 and 2009. **A:** Prevalence of overweight and obesity (BMI = or >25), Thai population age 18-59, NHES 1-4. **B:** Prevalence of diabetes, Thai population age 18-59, NHES 1-4.

Data Source: [32-35]

Despite high level and equitable access to care, effective coverage, measured by proportion of well control of key NCD such as hypertension and diabetes was low. Of those whose physical examination found hypertensive (systolic and diastolic blood pressure > 140 and > 90 mmHg), 79% and 61% among men and women were not diagnosed in 2004, a small proportion who were diagnosed but was not on treatment. The proportion of well controlled hypertension was 6% and 12% among men and women in 2004; however, in 2009 this proportion increased to 14% and 27% respectively, while the proportion of un-diagnosed reduced significantly as a result of hypertension screening campaign (Figure S11).


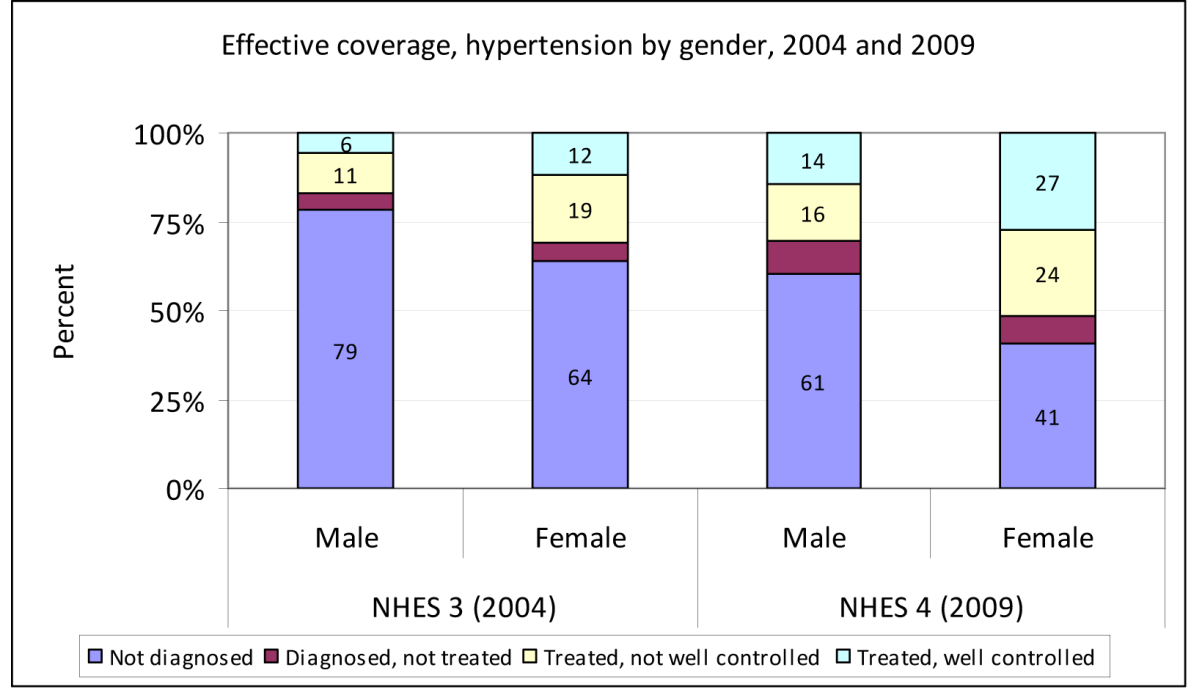


**Figure S11:** Effective coverage, hypertension by gender, 2004 and 2009

Data Source: [34-35]

Similarly, for those whose blood sugar tests were found as diabetes in the NHES (fasting plasma glucose above 140 mg/dL), 66% and 49% among men and women were not diagnosed in 2004, the proportion of well control diabetes was 9% and 15%. In 2009, the proportion of non-diagnosed diabetes reduced to 43% and 22% in men and women while the proportion of well controlled diabetes increased to 20% and 35% in men and women, Figure S12.


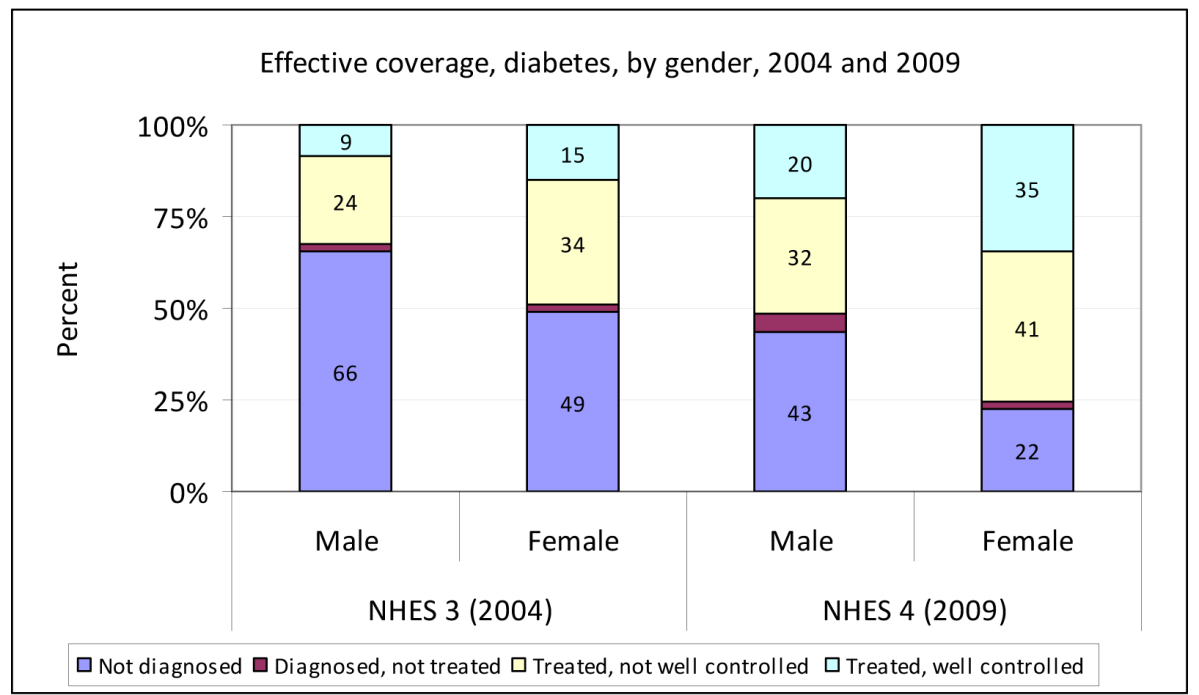


**Figure S12:** Effective coverage, diabetes by gender, 2004 and 2009

Data Source: [34-35]

Increased timely access to quality diagnostics and interventions resulted in improvement in certain key hospital treatment outcomes. The national hospitalization databases in 2005-2011 revealed a decreasing trend in case fatality among inpatients admitted with acute myocardial infarction, regardless of measures of the mortality: on arrival, at hospital discharge within 30 days after the admission (Figure S13).


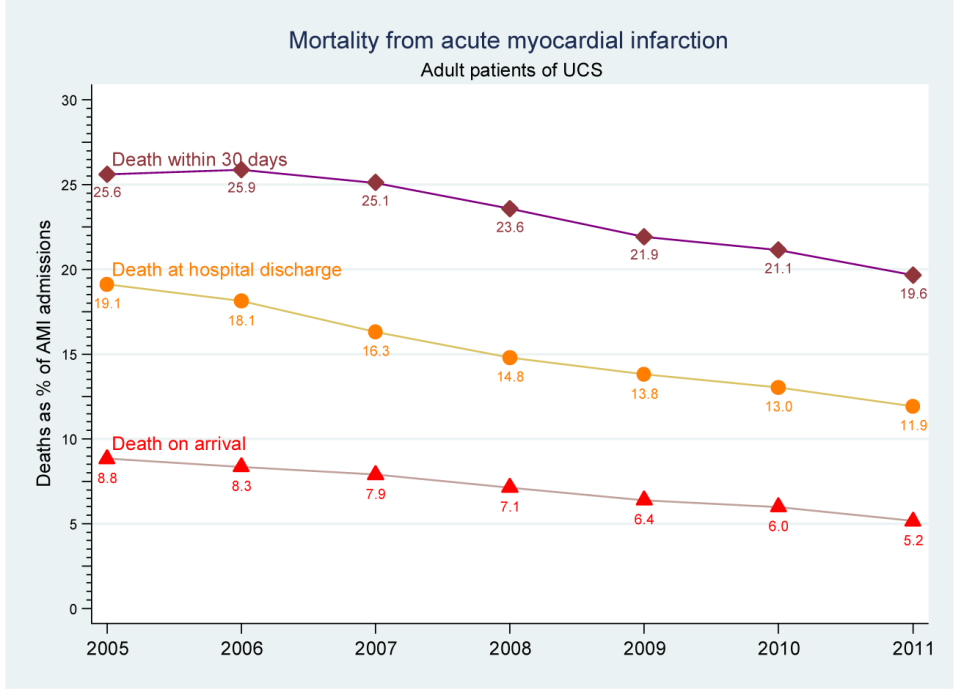


**Figure S13:** Reduced case fatality of patients hospitalized with acute myocardial infarction, 2005-2011

**5. Conclusions and recommendations**

Successful UHC outcome in Thailand are multi-factorial, functioning primary health care at DHS and availability of quality health service are important foundations for UHC implementation. Extensive benefit package and free at point of services result in high level of financial risk protection.

***Population coverage***

Since Civil Registry covered 96.7% and 95.2% of total births and deaths in 2005 Survey of Population Changes by NSO **[38]**, the completeness of insurance registry held by three insurance schemes was more than 95% of Thai citizens. Since the exact number of UCS registry, around 47 million was used for budget approval through annual Budget Bill, the accuracy and completeness was much improved. The Civil Registry and three schemes' member datasets were fully computerized, inter-operable and shared using the unique citizen ID number. For example, all births were daily registered to UCS or as child dependant of CSMBS (SHI did not cover dependants), the unemployed SHI members were automatically transferred to UCS, where as a UCS member, once employed will be transferred to SHI. Once the age of CSMBS child dependants beyond legal age of 20 years old, they would be transferred to UCS or SHI member if employed. The daily sharing of births and deaths, and updating membership across schemes resulted in real time accuracy ensuring entitlement to health benefits by members, as all healthcare providers can access to membership database via internet.

***Household surveys: how they were strengthened?***

Since 2002, there was no new national household surveys specifically designed for UHC monitoring, but built on and strengthened the existing NSO regular surveys. Monitoring of financial risk protection and equity in utilization was possible owing to the existence of SES **[39]**, and continued improvement of survey questionnaires.

Trust and mutual recognition between health reformists and NSO gradually built up since 1990s. Normally HWS was conducted every five year interval; in response to IHPP requests, NSO in 2002 decided to conduct HWS for five consecutive years between 2003 and 2007 for close monitoring impact on equity in utilization and health expenditure by households; and thereafter every two years in 2009, 2011 and 2013.

Upon IHPP requests in 1990s, NSO decided to insert a small module, on housing characteristics and ownership of durables, into the questionnaires of all health-related surveys which facilitated computation of wealth index as a key economic stratefier. This module is simpler, less time consuming in household interviews compared to income or expenditure questions.

The low incidence of catastrophic health expenditure can be misleading if the poor household decided not to seek care when ill, there was no expenditure on health and no catastrophic spending, but welfare loss such as mortality or disability if care were not timely given. For this reason, IHPP in collaboration with NSO introduced a standard question on unmet healthcare need using OECD method **[40]** into the HWS; unmet need was found very low, on par with OECD countries **[8]**.

***Routine data: how they were developed?***

Universal ART prompted the NHSO to developed ART registries with confidential protection; ART registry was most invaluable in monitoring mortality outcome given different level of CD4 count at entry by linking with the Civil Registration. Also universal renal replacement therapy prompted NHSO to establish End Stage Renal Disease (ESRD) patient registries to monitor mortality between peritoneal and hemo-dialysis and across Schemes. When the numbers of living ESRD patients and People Living with HIV/AIDS on ART were used for budget approval, the reliability was much improved.

While the unmet healthcare need generated from household survey was useful for broad monitoring, there is no national level dataset on waiting time of selected conditions such as cataract lens, hip and knee replacements; though primary data was recorded in the individual hospitals' logbooks for queuing and service management **[41]**.

***National ownership, institutional capacities and sustained M&E platforms***

HWS, equivalent to Demographic and Health Survey (DHS), was first conducted by NSO in 1970s, well before the advent of DHS in 1984. The recent introduction of unmet health care needs had proved useful for M&E. With strong collaboration between users and producers, these surveys had been very useful.

National Health Examination Surveys, locally initiated since 1991 **[32]**, financed by domestic resources had been able to sustain to the current fifth survey in 2013. The survey was responsible by National Health Examination Survey Office in collaboration with relevant partner institutes. NHES contributed to the understanding of low effective coverage of key NCD interventions and prompted active screening and control.

Locally initiated, financed and ownership of Burden of Diseases program since 1997, and NHA since 1994 **[26]** in IHPP, had been able to sustain to date. Locally initiated, financed and independent from donor driven agendas and resources ensured sustainability, flexibility on survey interval, continued improvement and use for policy decision in line with national interests. Several factors contribute to these successes, individual and institutional capacities to generate evidence in influencing policy formulation and systems design, monitoring and evaluation and effective feedback loop for adjustment; economic growth and improved fiscal space benefiting UC scheme through political and financial commitments; implementation capacities and supply side resilience to accommodate significant increases in service utilization.

***Recommendations***

Epidemiological transition towards chronic NCD and cost pressure from long term treatment warrant investment in primary preventions addressing proximal and distal determinants of ill health with the application of effective population based strategies making healthy behaviour a social norm **[42]**; rather than a conventional approach of early detection and prompt treatment, secondary prevention of complications. This requires multi-sectoral actions, beyond the command and control of the MOPH and ensures health in other sectoral policies such as national and international trades in tobacco, alcohol and soft drink, transport and road safety, food safety and carcinogen contaminations.

It light of this review, it is recommended to continue sustaining and strengthening M&E data platforms. Though the existing SES and HWS are good enough in measuring financial risk protection and equity in utilization, they had limitation in measuring effective coverage for which bio-markers are required, such as diabetes and hypertension as covered by NHES. However, NHES was less frequently conducted (every five years) and not timely to meet policy uses. Self-reported unmet healthcare needs embedded into NSO surveys are crude but useful indicator for broad monitoring, however it was subject to differences in expectation by rich and poor individuals. The M&E system has yet to develop a waiting time of key elective surgeries **[41]**; though currently was recorded in hospital level log books with great variations in definition. Waiting time is useful for benchmarking and improvement.

Maintaining high coverage of Civil Registration--essential for counting all births and deaths and ensuring the rights to health, is as important as improved accuracy of cause of deaths for measuring outcome of interventions. Challenges are a majority, 60% of total annual mortalities took place at home, where lay-diagnosed cause of death was provided; this results in high level of inaccuracy **[43]**.

Routine health facilities electronic data are useful for monitoring the variations in treatment outcome by public and private providers and insurance schemes. Success in establishing a single national inpatient dataset consisting of inpatients in three schemes has yet to extend to outpatient and specific diseases registries, such as the current fragmented ART and RRT M&E systems. See Box S4.

| **Box S4: Recommendations**  1. Two main M&E data platforms, representative household surveys and health facility electronic databases should be sustained and strengthened.  2. Measures the overall level and distribution of health utilization and financial risk protection through the use of SES and HWS should be complement by measures of the effective coverage of health care through the use of NHES.  3. Concerning responsiveness to people expectation of the UHC, the M&E data have yet to contain measures of a waiting time of health services such as key elective surgeries.  4. Maintaining high coverage of Civil Registration is as important as improved accuracy of cause of deaths for measuring outcome of interventions.  5. Routine health facilities electronic data are useful for monitoring the variations in treatment outcome by public and private providers and insurance schemes. Success in establishing a single national inpatient datasets need to extend to outpatient and specific diseases registries. |
| --- |

**Acknowledgments**

Initiations by National Statistical Office especially SES since 1950s and HWS since 1970s, and its evolution for monitoring UHC progresses, and routine administrative facility based information system by NHSO and partners were recognized.

Special thanks are going to Dr. Ties Boerma, Dr David B Evans, Dr. Carla Abouzahr and Dr. Priyanka Saksena for their technical discussion and comments. We also wish to acknowledge inputs on UHC by the late Guy Carrin and the late Sanguan Nitayarumphong.

**Abbreviations**

| **ART** | Anti-Retroviral Therapy | | |  |  |  |  |
| --- | --- | --- | --- | --- | --- | --- | --- |
| **CSMBS** | Civil Servant Medical Benefit Scheme | | |  |  |  |  |
| **DHS** | District Health System | | |  |  |  |  |
| **HWS** | Health and Welfare Survey | | |  |  |  |  |
| **M&E** | Monitoring and Evaluation | | |  |  |  |  |
| **MICS** | Multi-Indicator Cluster Survey | | |  |  |  |  |
| **MOPH** | Ministry of Public Health | | |  |  |  |  |
| **NHES** | National Health Examination Survey | | |  |  |  |  |
| **NHSO** | National Health Security Office | | |  |  |  |  |
| **OECD** | Organization for Economic Co-operation and Development | | |  |  |  |  |
| **OOP** | Out-of-Pocket | | |  |  |  |  |
| **PMTCT** | Prevention of Maternal to Child HIV Transmission | | |  |  |  |  |
| **SES** | Socio-Economic Survey | | |  |  |  |  |
| **SHI** | Social Health Insurance | | |  |  |  |  |
| **UCS** | Universal Coverage Scheme | | |  |  |  |  |
| **UHC** | Universal Health Coverage | | |  |  |  |  |
| **DIMENSIONS** | **INDICATORS** | **DATA PLATFORMS** | **FREQUENCY** | **RESPONSIBLE AGENCIES** | **LEVEL** | **DISTRIBUTION** | **POLICY USES** |
| **INPUTS** | **1. Financing**   - THE, % GDP - GGHE, % GGE - THE per capita - OOP, % of THE - Total HIV/AIDS expenditure, % THE | - Socio-Economic Survey (SES) - National Health Account - National AIDS Spending Assessment | - Biannual until 2008, then annual - Annual NHA since 1994 - NASA: biannual since 2000 | - National Statistical Office - IHPP for NHA and NASA | Yes, | No | Monitor and long term 20 years projection of THE as % GDP, monitor HIV/AIDS program expenditure as part of UNGASS report every two years |
|  | **2. Infrastructure and health workforce**   - Health facility per 1000 pop - Hospital bed per 1000 pop - Doctor per 1000 pop - Nurse and midwives per 1000 pop | - MOPH annual Health Resource Survey | Annual survey since 1980s | MOPH | Yes | By province, urban and rural | Equity monitoring, investment and health workforce allocation decision |
|  | **3. Medicines in CSMBS OP**   - non-essential medicines, % of total items and value dispensed | - CSMBS OP prescription database in 34 hospitals, covering approximately half of total OP reimbursement expenditure | Real time upon claim submission | Comptroller General Department through Central Health Information Office | Yes | No | Monitor by CGD |
| **OUTPUTS** | **1. Population coverage**   - Number of population coverage by insurance fund | - Civil Registration [high coverage 96.7% for all birth, 95.2% for all deaths] | - Daily update by MOI Civil Registration Bureau, - Weekly linked with membership registration dataset by 3 insurance schemes | - Civil registration Bureau, - NHSO as focal point of 3 schemes | Yes | By 3 insurance schemes, demographic profiles (age, gender) | Ensuring all citizens are covered, and seamless transfer from one to another as citizen entitlement for health care |
|  | **2. Utilization and profiles**   - OP visit per capita, - Admission rate per capita, - OP/IP use profile: public, private, level of care (primary, secondary, tertiary) - Unmet healthcare needs, % total needs (OECD standard questionnaire) - Contraceptive prevalence rate - Adolescent unmet family planning services, % | - Health and Welfare Survey (HWS) - Other NSO regular national representative household surveys - 3 Health Insurance Scheme throughput datasets e.g. OP, IP, high cost care | - HWS: every five years until 2001, then annual between 2003 and 2007, Biannual thereafter, 2009, 2011, 2013 - Others: Elderly Survey every five years, Disable survey, every five years, Reproductive Health Survey, every five years - MICS: 2006 and 2012 | - NSO - NSO and UNICEF for MICS | Yes | By wealth quintiles, urban rural, maternal education | Ensuring equitable utilization and prioritize specific intervention to improve access, unmet healthcare need vital information to monitor progresses towards UHC |
|  | **3. Service quality and safety**   - Accredited health facilities accredited by 3 stages: 1^st^ step, 2^nd^ step, accredited, re-accredited, % total - TB treatment success rate, % - 30 day hospital case fatality rate acute myocardial infarction, stroke - Waiting time elective surgery: cataract, hip replacement - Surgical wound infection,  % total clean surgeries | - Accreditation status certified by Healthcare Accreditation Institute (HAI), | - Re-accreditation required every three years - Step 1 and 2 of quality improvement valid for 3 years | HAI | Yes | By type of providers, primary healthcare centres, public and private hospitals, | Quality improvement strategies, hospital self monitoring, risk management and prevention, financial incentives by NHSO in favour of accredited status, than Step 2 and 1 respectively |
| **OUTCOME** | **1. Service coverage**   - Quality four ANC visits, % total - Skill birth attendants, institutional births, % total - DTP3 and measles coverage, % children <1 - Contraceptive prevalence rate and profiles - PMTCT coverage, % eligible HIV positive pregnancies - ART coverage, % eligible adults, children - Coverage of renal replacement therapy | - HWS - MICS - Special programs databases: National AIDS program, Renal Replacement Therapy, Pap-smear, Influenza vaccine | - HWS biannual - 2 round MICS, 2006 and 2012) - Routine NHSO admin dataset and specific disease registries such as ART, Dialysis | - NSO - NSO/UNICEF - NHSO | Yes | By wealth quintiles, maternal education, urban/rural, | Use for annual budget allocation and monitor progresses |
|  | **2. Financial risk protection**   - OOP, % THE - Incidence of catastrophic health expenditure - Incidence of impoverishment | - SES - National poverty line | - Annual SES - Regular update urban/rural poverty lines | - NSO - NESDB | Yes | By wealth quintiles, urban/rural | Monitor progresses on financial risk protection, |
|  | **3. Benefit Incidence**   - Concentration index -1 to + 1 | - SES - HWS - Unit cost | - SES: annual - HWS: biannual - Unit cost: infrequent research studies | - NSO - Research institute | Yes | By wealth quintiles, urban/rural | Monitor progresses on financial risk protection, |
| **IMPACTS** | **1. Improved health**   - Effective coverage of DM and HT: % knowing of having the disease, % under treatment, % well control - Disease specific mortality rates - Survival curve of specific diseases: end stage renal patients under renal replacement therapy | - National Health Examination Survey (NHES, - MICS (2006, 2012) - MOI Civil Registry linked with national IP dataset - Specific disease registries: RRT, Thalassemia | - Four waves: 1990, 1997, 2004 and 2009 - MICS 2006, 2012 - Daily update of vital events in Civil Registration - Routine updates | - HSRI for NHES - NSO/UNICEF for MICS - MOI Civil Registration Bureau - Three insurance scheme patient IP dataset - Kidney Foundation, and NHSO for disease registries | Yes | - By wealth quintiles, urban/rural, insurance schemes - Survival of hemo- versus peritoneal dialysis and specific cancers by three insurance scheme, | Monitor health outcome of different interventions, across socio-economic profiles and insurance schemes, inadequate effective coverage from NHES results in special program on DM and HT |
|  | **2. Increased responsiveness**   - % satisfaction to UCS by members, and healthcare providers - % IP reported being treated badly by health staffs on confidentiality, prompt attention, communication and information, respectful treatment with dignity, with the application of vignettes for standardization - % OP and IP satisfied with hospital services | - Independent Poll monitoring (ABAC 2011, latest) - Responsiveness Survey (HWS 2013) - OP and IP surveys by hospitals - Call centre data | - Poll survey: annual - HWS: biannual - Hospital OP / IP surveys - Call centre annual report | - NHSO for annual poll surveys - NSO for HWS - Hospital survey: : ad hoc - NHSO for complaining report | Yes | By wealth quintiles, urban/rural for HWS | For quality and responsiveness self improvement, national and local monitoring and uses |

**Table S2**: National framework of sets of Indicators, data platforms and policy uses for UHC measurement, Thailand 2013

Data source: Authors’ synthesis

**References**

1. Srithamrongsawat S, Wisessang R, Ratjaroenkhajorn S. Financing healthcare for migrants; a case study from Thailand. International Organization for Migration and World Health Organization 2009

2. Jongudomsuk P., Limwattananon S., Prakongsai P., Srithamrongsawat S., Pachanee K., Mohara A., Patcharanarumol W., and Tangcharoensathien V. Evidence-based health financing reform in Thailand (chapter 16). In Clements B., Coady D., and Gupta S., eds. The Economics of public health care reform in advanced and emerging economies, 307-26. Washington, DC : International Monetary Fund, 2012.

3. Hu S, Tang S, Liu Y, Zhao Y, Escobar ML, de Ferranti D, Reform of how health care is paid for in China: challenges and opportunities. Lancet, 2008; DOI:10.1016/S0140-6736(08)61368-9.

4. Yip W, Hsiao W, Meng Q, Chen W, Sun X. Realignment of incentives for health-care providers in China. Lancet 2010; 375: 1120–30.

5. World Health Organization. Health systems financing: the path to universal coverage. World health report 2010. Geneva, 2010.

6. Evans TG, Chowdhury AMR, Evans D, Fidler A, Lindelow M, Mills A, and Scheil-Adlung X, Thailand’s Universal Coverage Scheme: Achievements and Challenges. An Independent Assessment of the First 10 Years (2001-2010) (Nonthaburi, Thailand: Health Insurance System Research Office, 2012.

7. Tangcharoensathien V., Pitayarangsarit S., Patcharanarumol W., Prakongsai P., Sumalee H., Tosanguan J., and Mills A. Promoting universal financial protection: how the Thai universal coverage scheme was designed to ensure equity. Health Research Policy and Systems 2013;11:25.

8. Thammatacharee N., Tisayaticom K., Suphanchaimat R., Limwattananon S., Puthasri W., Netsaengtip R., and Tangcharoensathien V. Prevalence and profiles of unmet need in Thailand. BMC Public Health 2012; 12: 923.

9. Limwattananon S., Tangcharoensathien V., and Prakongsai P. Catastrophic and poverty impacts of health payments: results from national household surveys in Thailand. Bulletin of the World Health Organization 2007; 85: 600–6.

10. Rohde J, Cousens S, Chopra M, Tangcharoensathien V, Black R, Bhutta Z, Lawn JE. Alma-Ata: Rebirth and Revision 4, 30 years after Alma-Ata: has primary health care worked in countries? Lancet 2008; 372: 950–61.

11. World Bank, World Development Indicators: Contraceptive prevalence rate (% of women age 15-49) available at <http://data.worldbank.org/indicator/SP.DYN.CONU.ZS> [access 3 October 2013]

12. Kongsri S., Limwattananon S., Sirilak S., Prakongsai P., and Tangcharoensathien V. Equity of access to and utilization of reproductive health services in Thailand: national reproductive health survey data, 2006 and 2009. Reproductive Health Matters 2011; 19: 86–97.

13. National Statistical Office, Survey of Population Changes, various years: 1975, 1985, 1995 and 2005. Nonthaburi: Ministry of Information, Communication and Technology.

14. Bundhamcharoen K, Odton P, Phulkerd S, Tangcharoensathien V. Burden of disease in Thailand: changes in health gap between 1999 and 2004 BMC Public Health. 2011; 11:53.

15. Aungkulanon S, McCarron M, Lertiendumrong J, Olsen SJ, Bundhamcharoen K. Infectious disease mortality rates, Thailand, 1958-2009. Emerg Infect Dis 2012 Nov; 801-1794: (11)18.

16. Analysis from World Health Statistics: 2000-2005

17. Rajaratnam JK, Marcus JR, Levin-Rector A, Chalupka AN, Wang H, Dwyer L, Costa M, et al Worldwide mortality in men and women aged 15–59 years from 1970 to 2010: a systematic analysis. Lancet 2010; on line publication, April 30, 2010, DOI:10.1016/S0140-6736(10)60517-X

18. National Economic and Social Development Board. Thailand Millennium Development Goal Report 2004, Bangkok, 2004., p. 34.

19. Waage F., Banerji R., Campbell O., Chirwa E., Collender G., Dieltiens V., Dorward A., Godfrey-Faussett P., Hanvoravongchai P., Kingdon G., Little A., Mills A., Mulholland K., Mwinga A., North A., Patcharanarumol W., Poulton C., Tangcharoensathien V., and Unterhalter E. The Millennium Development Goals: a cross-sectoral analysis and principles for goal setting after 2015: Lancet and London International Development Centre Commission. The Lancet 2010; 376: 991-1023.

20. Putthasri W., Suphanchaimat R., Topothai T., Wisaijohn T., Thammatacharee N., and Tangcharoensathien V. Thailand special recruitment track of medical students: a series of annual cross-sectional surveys on the new graduates between 2010 and 2012. Human Resources for Health 2013;11:47.

21. Tangcharoensathien V., Prakongsai P., Limwattananon S., Patcharanarumol W., and Jongudomsuk P. From targeting to Universality : lessons from the health system in Thailand (Chapter 16). In Peter Townsend, editor. Building decent societies : rethinking the role of social security in development, 310-22. Houndmills, Basingstoke, Hampshire : Palgrave Macmillan, 2009.

22. Limwattananon S., Tangcharoensathien V., Tisayathicom K., Boonyapaisarncharoen T., and Prakongsai P. Why has the universal coverage scheme in Thailand achieved a pro-poor public subsidy for health care? BMC Public Health 2012; 12 (suppl 1): S6.

23. Patcharanarumol W., Tangcharoensathien V., Limwattananon S., Panichkriangkrai W., Pachanee K., Poungkantha W., Gilson L., and Mills A. Why and how did Thailand achieve good health at low cost? (chapter 7). In Balabanova D., McKee M., and Mills A., eds. ‘Good health at low cost’ 25 years on. What makes a successful health system? 193-223. London : London School of Hygiene & Tropical Medicine, 2011.

24. Ministry of Public Health. Key performance indicators, fiscal year 2012. Available at <http://healthdata.moph.go.th/kpi/2555/KpiTopicList.php> [access 5 October 2013]

25. National Statistical Office, Health and Welfare Survey 2011. Nonthaburi, Ministry of Information and Communication Technology, 2012

26. Tangcharoensathien V., Laixuthai A., Vasavit J., Tantigate N-A., Prajuabmoh-Ruffolo W., Vimolkit D., and Lertiendumrong J. National health account development: lessons from Thailand. Health Policy and Planning 1999; 14: 342-53.

27. Patcharanarumol W, Cichon M, Tangcharoensathien V, Vasvid C, Tisayaticom K. Research Series of Thai Health Care Financing: Part 1 Financial Reform Options of Health Care Coverage in Thailand. Journal of Health Science 2006; 15:17-30.

28. Sakunphanit, T. et.al. 2009. Trend of cost and service in Thai health delivery systems. Preliminary report

29. Healthcare Accreditation Institute, Thailand. available at <http://www.ha.or.th/haweb/index.php/home-2> [access 3 September 2013]

30. Thamarangsi T. Addiction research centres and the nurturing of creativity: Center for Alcohol Studies (CAS), Thailand Addiction, 108, 1201–1206. doi:10.1111/j.1360-0443.2012.03795.x

31. Tangcharoensathien V., Limwattananon S., and Prakongsai P. Improving health-related information systems to monitor equity in health: lessons from Thailand. In Di McIntyre, and Gavin Mooney, eds. The Economics of health equity, 222-46. New York: Cambridge University Press, 2007.

32. Choprapawan C, editor. The First National Health Examination Survey 1991-1992. Bangkok: National Epidemiology Board of Thailand and Thailand Health Research Institute, 1992.

33. The Technical Committee for National Health Examination Survey II. The Second National Health Examination Survey in Thailand 1996-1997. Bangkok: Health System Research Institute, 1998.

34. Porapakham Y and Bunyaratapun P, Editors. The Third National Health Examination Survey 2003-4. Bangkok: War Veterans Organization Printing, 2006.

35. Aekpalakorn, W. et al. The Fourth National Health Examination Survey 2008-9. the National Health Examination Survey Office, Health System Research Institute. 2010.

36. Health expenditure and financing in OECD countries, 2010. Available at <http://stats.oecd.org/index.aspx?DataSetCode=HEALTH_STAT> (access 19 December 2012)

37. Gruber J, Hendren N, Townsend RM. The Great Equalizer: Health Care Access and Infant Mortality in Thailand. American Economic Journal: Applied Economics, 2014; 6(1): 91-107.

38. National Statistical Office. Report on The 2005-2006 Survey of Population Change. Available at <http://www.ocsc.go.th/ocsc/th/files/BRD_Reserch/thesis%20knowledge/May_2013/50005.pdf> [access 5 October 2013]

39. National Statistical Office. History of Socio-Economic Survey. Available at <http://sisaket.nso.go.th/sisaket/sss/sss.html> [access 20 October 2013]

40. de Looper M, Lafortune G: Measuring Disparities in Health Status and in Access and Use of Health Care in OECD Countries. In OECD Health Working Papers No. 43. Paris: OECD publishing; 2009.

41. Viberg N, Forsberg B, Borowitz M, Molin R. International comparisons of waiting times in health care, limitations and prospects. Health Policy, 2013; 112 : 53– 61

42. WHO, The world health report 2002: Reducing Risks, Promoting Healthy Life. Geneva, World Health Organization, 2002

43. Tangcharoensathien V., Faramnuayphol P., Teokul W., Bundhamcharoen K., and Wibulpolprasert S. A Critical assessment of mortality statistics in Thailand: potential for improvements. Bulletin of the World Health Organization 2006; 84: 233-8.
